# Penile secondary lesions: a rare entity detected by PET/CT

**DOI:** 10.1038/s41598-021-85300-8

**Published:** 2021-03-15

**Authors:** Tima Davidson, Liran Domachevsky, Yogev Giladi, Eddie Fridman, Zohar Dotan, Barak Rosenzweig, Raya Leibowitz, Jennifer Ben Shimol

**Affiliations:** 1 Department of Nuclear Medicine, Sheba Medical Center, Derech Sheba 2, 52621 Ramat Gan, Tel-Hashomer, Israel; 2 Sackler Faculty of Medicine, Tel-Aviv University, 6997801 Tel-Aviv, Israel; 3 Department of Pathology, Sheba Medical Center, 52621 Tel Hashomer, Israel; 4 Department of Urology, Sheba Medical Center, 52621 Tel Hashomer, Israel; 5 Oncology Institute, Shamir Medical Center, 70300 Zerifin, Israel; 6 Department of Medicine, E. Wolfson Medical Center, 5822012 Holon, Israel

**Keywords:** Cancer imaging, Penile cancer, Prostatic diseases

## Abstract

While penile metastases are rare, PET/CT has facilitated their detection. We aimed to describe penile secondary lesions (PSL) identified by PET/CT. We reviewed 18F-FDG and Ga68-PSMA PET/CT records performed in a single center during May 2012-March 2020, for PSL. Of 16,774 18F-FDG and 1,963 Ga68-PSMA-PET scans, PSL were found in 24(0.13%) men with a mean age of 74. PSMA detected PSL in 12 with prostate cancer; FDG identified PSL in 4 with lymphoma, 3 with colorectal cancer, 2 with lung cancer, and one each with bladder cancer, pelvic sarcoma, and leukemia. Mean SUVmax of PSL was 7.9 ± 4.2 with focal uptake in 13(54%). Mean lesion size was 16.5 ± 6.8 mm; 8 at the penile root, 4 along the shaft, and 1 at the glans. CT detected loss of the penile texture in 15(63%). PSL were observed only during relapse or follow-up of disseminated disease. Among those with prostate cancer, PSA varied widely. Fifteen (62.5%) died, at a mean 13.3 ± 15.9 months following PSL demonstration, nine had non-prostate malignancies. PET/CT identified and characterized PSL in a fraction of cancer patients, most commonly those with prostate cancer. PSL universally surfaced in advanced disease, and signaled high mortality, especially in non-prostate cancers.

## Introduction

Penile secondary lesions (PSL) appear infrequently among cancer patients; several hundred cases have been reported since Eberth originally described them in 1870^1–5^. Most are seen in cancers of the genitourinary and lower gastrointestinal tracts; 29% were reported to originate in the bladder, 28% in the prostate, and 12% in conjunction with rectosigmoid cancer^5^.

Clinical features of PSL include palpable nodules, ulcerations, obstructive or irritative urinary symptoms, hematuria, and priapism, often with significant morbidity^3^. PSL may originate from venous spread, lymphatic invasion, or local infiltration. While the rich arterial and venous communication in the penile region typically limits tumor cell seeding, venous or lymphatic occlusion from neighboring cancers may enable implantation^5,6^.

PSL detection is limited in most imaging modalities, and lesions are likely missed when asymptomatic, which occurs in up to 12%, or when the symptoms are attributed to the primary cancer. CT and MRI do not routinely capture the penis. These modalities also do not adequately detect occult involvement of normal-sized lymph nodes^7–11^. With ultrasound, PSL may be obscured by the pubic bone or pubic fat pad, or from attenuation caused by the flaccid tunica albuginea^12^.

The increasing use of PET/CT to stage malignancies, by means of 18F-FDG (18-fluorodeoxyglucose) and Ga 68-PSMA (gallium 68-labeled prostate specific membrane antigen), facilitates the detection of PSL^13–17^. However, to our awareness, there are no published papers describing these PET/CT findings in a series of patients with multiple types of malignancies. Our objective was to characterize the PET/CT features of PSL in cancer patients at a large tertiary institution.

## Methods

### Ethics

This single-institution study was approved by the institutional review board of Sheba Medical Center, according to the Declaration of Helsinki (approval no: SMC-20-7097). Informed consent was waived by the institutional review board of Sheba Medical Center due to the retrospective nature of the study.

### Study design

We searched the computerized database of Sheba Medical Center, a tertiary hospital, for PET/CT imaging reports from May 2012 to March 2020 that included the term “penis” or “penile”. Once identified, relevant reports and images were individually reviewed to confirm the presence of an FDG- or PSMA-avid penile lesion. Imaging data were retrieved from the picture archive and communication system (PACS, Carestream Health 11.0, Rochester, NY), and clinical data were obtained from the computerized medical records at our hospital. Clinical data, including medical history, laboratory work, and biopsy results were reviewed.

Study inclusion criteria were: (1) men aged 18 years and older; (2) a history of a malignancy with a primary site that was non-penile; and (3) findings consistent with PSL on PET/CT. Exclusion criteria included the presence of abnormal findings in the genital area on PET/CT. These included urinary retention in the penile urethra, without clear demonstration of a suspicious penile lesion, and evidence of radiotracer contamination in the penile area.

This retrospective study evaluated consecutive patients. During the designated period, a total of 48,731 18-F-FDG PET/CT scans were performed, mainly for assessment of known or suspected oncological diseases; of them, 22,875 scans were performed on 16,774 unique male patients. From 2015 onward, PET/CT using Ga 68-PSMA was performed in men with diagnosed or suspected prostate cancer or with biochemical recurrence. In total, 2,087 Ga 68-PSMA scans were carried out on 1,963 men. From a total of 18,737 unique PET/CT studies, the reports of 57 men mentioned the word “penis” or “penile”, and 24 (0.13%) met the study eligibility criteria. Of the 1963 men with Ga 68-PSMA studies, 12 (0.61%) met the eligibility criteria. The remaining were excluded due to the absence of findings compatible with PSL.

### PET/CT image acquisition

PET/CT scanning was performed using a combined PET-CT protocol with a 16-detector-row helical CT scanner (Gemini GXL, Phillips Healthcare). This scanner enables simultaneous acquisition of up to 45 transaxial PET images, with interslice spacing of 5 mm in one bed position; and provides an image from the vertex to the thigh in about 10 bed positions. The transaxial fields of view and pixel sizes of the PET images reconstructed for fusion were 57.6 cm and 4 mm, respectively, with a matrix size of 144 × 144 mm. The CT component was performed in accordance with the hospital’s standard protocol, with routine use of both oral and intravenous contrast media unless either was contraindicated. The following technical parameters were used for CT imaging: pitch 0.8, gantry rotation speed 0.5, 120 kVp, 250 mAs, 3-mm slice thickness, and specific breath-holding instructions^18–20^.

Depending on the type of PET/CT scan (with FDG or PSMA), the patient received an intravenous injection of 370 MBq 18F-FDG after 4–6 h of fasting, or an injection of 148 MBq Ga 68-PSMA in the absence of a prerequisite fast. About 60 min later, CT images were obtained from the vertex to the mid-thigh for about 32 s. A contrast-enhanced CT scan was captured 60 s after injection of 2 mL/kg of non-ionic contrast material (Omnipaque 370 GE Healthcare). An emission PET scan followed in 3D acquisition mode for the same axial image range, 2.0–2.5 min per bed position. The diagnostic CT images were used for fusion with the PET data and to produce a map for attenuation correction. PET images were generated with CT attenuation correction utilizing a line of response protocol, and the reconstructed images were constructed for review (EWB, Extended Brilliance Workstation, Philips Medical Systems, Cleveland OH, USA)^18–20^.

### Image interpretation

All available images were interpreted by experienced specialists in nuclear medicine and radiology, and re-reviewed by one of the study co-authors with 20 years’ experience and dual certification in radiology and nuclear medicine. Readers were not blinded to clinical information. Consensus was achieved on interpretation of all of the images.

Ga 68-PSMA and 18F-FDG activity were quantified by calculating a maximum standardized uptake value (SUVmax). This was done by manually generating a region of interest over the sites of abnormally increased radioactive material activity. Increased Ga 68-PSMA/ 18F-FDG uptake that was not explained by the normal bio-distribution, or uptake that was higher than the physiological uptake in the surrounding tissue was considered pathological/positive. Positive PET findings were analyzed with respect to their intensity of uptake (SUV max) and pattern of uptake enhancement (focal or diffuse). Cases with more than 2 focal penile findings were considered diffuse.

CT images were examined for abnormalities of the penis that corresponded to PET findings, and classified as loss of the typically smooth penile texture. The following descriptions were used: a focal hyperdense soft tissue mass, a focal hypodense lesion, a diffuse heterogeneous soft tissue mass infiltration along the penis, or the absence of detectable CT findings. For focal lesions, the diameter length was measured and the location within the penis was evaluated, and labeled as: at the root, along the shaft, or at the glans.

We also examined the involvement of other structures within the pelvis (prostate, prostatic bed after prostatectomy, seminal vesicles, bladder, pelvic lymph nodes) and organs outside the pelvis.

We analyzed penile findings on ultrasound and MRI when available. For men with more than one PET/CT scan, we assessed the dynamic changes in penile lesions with regard to size and the persistence of increased uptake in all the consecutive imaging studies.

### Clinical data extraction

Since prostate cancer was the most common primary cancer in this study, we assessed specifically the characteristics of the men who had this cancer. For this subgroup, we examined the history of prostate resection including the type of operation, the method of approach, the values of prostate specific antigen (PSA), and Gleason’s score of the primary tumor lesion.

### Statistical analysis

Data are represented as means ± standard deviations (SDs) for continuous variables and as percentages for categorical parameters. The Chi-square test, Fisher’s exact test, and the Student’s t-test were used for statistical comparison as appropriate. The analysis was performed with the use of SPSS version 21.0 (SPSS, IBM, USA).

## Results

### Features of disease

#### Baseline characteristics

The mean age of the men included in the study was 74 ± 12.1 years (range 49–95). Sixteen men (66.7%) had primary genitourinary and lower gastrointestinal cancers. Prostate was the most common primary site, comprising 12 (50%) cases; their mean age was 78 ± 9.8 years (range 62–95). Of the other malignancies, four (16.7%) were lymphoma, three (12.5%) colorectal cancer (CRC), two (8.3%) lung cancer, one bladder cancer, one pelvic sarcoma, and one leukemia.

Two men with lung cancer and one man with lymphoma also had histories of prostate cancer. One of the patients with prostate cancer had a history of bladder cancer. Primary cancers were determined for this study based on both review of the notes written by the treating oncologists and on the PET/CT appearance of the penile lesions compared with other malignant appearing lesions.

#### Morphology of prostate tumors and Gleason’s score

Histological classification was available for 11 of the 12 men with primary prostate cancer. These 11 patients showed cell histology characteristic of adenocarcinoma of the prostate. Further subtyping was available for only 3 men, two were categorized as having acinar histology and the third as ductal. Three of the tissue biopsies with adenocarcinomas showed perineural invasion. For one man whose prostate tissue revealed adenocarcinoma, a biopsy of an inguinal lymph node (LN) revealed two cell populations. One of these was consistent with carcinoma with neuroendocrine differentiation, and the other with morphology favoring prostatic origin. For the patient with prostate cancer and lymphoma, and for the patient with both prostate and lung cancers, biopsies showed adenocarcinoma without further subtyping listed. Gleason’s scores, as assessed from prostate biopsies, were available for 10 men, and ranged from 6 to 10, with a mean of 7.6 ± 1.2.

#### History of surgical prostate removal

Of the 12 men with primary prostate cancer, 6 (50%) underwent prostatectomy. Three (25%) underwent transurethral resection of the prostate (TURP). Two men underwent robot-assisted laparoscopic radical prostatectomy. The last patient underwent radical prostatectomy for which the exact approach was not available.

In addition, the patient with lymphoma and a history of prostate cancer had undergone radical retropubic prostatectomy. The patient with bladder cancer, whose primary tumor invaded into the prostate, underwent suprapubic prostatectomy followed by TURP. Finally, one of the patients with lung cancer and a history of prostate cancer had undergone TURP.

#### Morphology of non-prostatic tumors

In the patient with primary bladder cancer, cell histology from the bladder biopsy was consistent with high grade urothelial carcinoma (UC) with necrotic features. The patient with primary prostate cancer who also had a history of bladder cancer had high grade UC with squamous differentiation. In the two men with lung cancers, the cell histology of one was consistent with non-small cell lung cancer (NSCLC) undifferentiated subtype, while the other patient had adenocarcinoma of the lung. All 4 patients with lymphoma had diffuse large B cell lymphoma (DLBCL). The 3 men with CRC had adenocarcinoma. The man with pelvic sarcoma had a high grade leiomyosarcoma. The patient with leukemia had acute myeloid leukemia (AML).

A penile biopsy was performed in only one man in our cohort, one of the patients with CRC. The tissue histology revealed cellular features of colorectal adenocarcinoma.

### Findings on PET/CT

#### Penile lesions on PET

##### SUVmax of PSL

For the entire cohort, the mean SUVmax of the penile lesions was 7.9 ± 4.2, with a range of 2.5–18.2. For one patient with prostate cancer who was being treated with hormonal therapy, SUVmax at the penis was too low to be measured. The mean SUVmax for the remaining 11 men with prostate cancer was 6.8 ± 3.8, with a range of 2.5 to 17.2 (Table 1). No correlation was detected between cancer histology and the degree of tracer uptake (*p* = 0.240). In the subgroup of patients with prostate cancer, there was no significant difference in SUVmax between those who had undergone prostate resection and those who had not (*p* = 0.300).

**Table 1:** Characteristics of penile lesions seen on PET/CT according to primary malignancy.

Patient	Primary cancer type	Location of the penile lesion	Pattern of uptake enhancement	SUVmax	Penile findings on CT	Size of focal lesions (mm)
1	Prostate	Penile shaft	Focal	6.5	None	10
2	Prostate	Penile shaft	Focal	5.4	Hyperdense soft tissue mass	12
3	Prostate	Entire penis	Diffuse	4.2	Hyperdense soft tissue infiltration	
4	Prostate	Entire penis	Diffuse	Unmeasurable	Hyperdense soft tissue infiltration	
5	Prostate	Entire penis	Diffuse	2.5	Hyperdense soft tissue mass	
6	Prostate	Penile root	Focal	5.8	None	17
7	Prostate	Entire penis	Diffuse	17.2	Hyperdense soft tissue infiltration	
8	Prostate	Penile root	Focal	6.5	Hyperdense soft tissue mass	10
9	Prostate	Penile root	Focal	5.1	None	10
10	Prostate	Entire penis	Diffuse	7.4	Hyperdense soft tissue infiltration	
11	Prostate	Entire penis	Diffuse	7.8	Hypodense lesion	
12	Prostate	Entire penis	Diffuse	6.7	Hyperdense soft tissue infiltration	
13	Lymphoma	Penile glans	Focal	3.2	None	12
14	Lymphoma	Entire penis	Diffuse	8.2	None	
15	Lymphoma	Entire penis	Diffuse	8.4	Hyperdense soft tissue infiltration	
16	Lymphoma	Entire penis	Diffuse	17.2	None	
17	Colorectal	Entire penis	Diffuse	7.2	Hyperdense soft tissue infiltration	
18	Colorectal	Penile root	Focal	6.2	Hyperdense soft tissue mass	15
19	Colorectal	Penile root	Focal	10	None	30
20	Lung	Penile shaft	Focal	5.5	Hyperdense soft tissue mass	13
21	Lung	Penile shaft	Focal	7.8	Hypodense lesion	22
22	Bladder	Penile root	Focal	10.6	None	22
23	Pelvic leiomyosarcoma	Penile root	Focal	18.2	Soft tissue mass	28
24	AML	Penile root	Focal	4.8	None	15

##### The pattern of uptake of PSL

**Figure 1:**
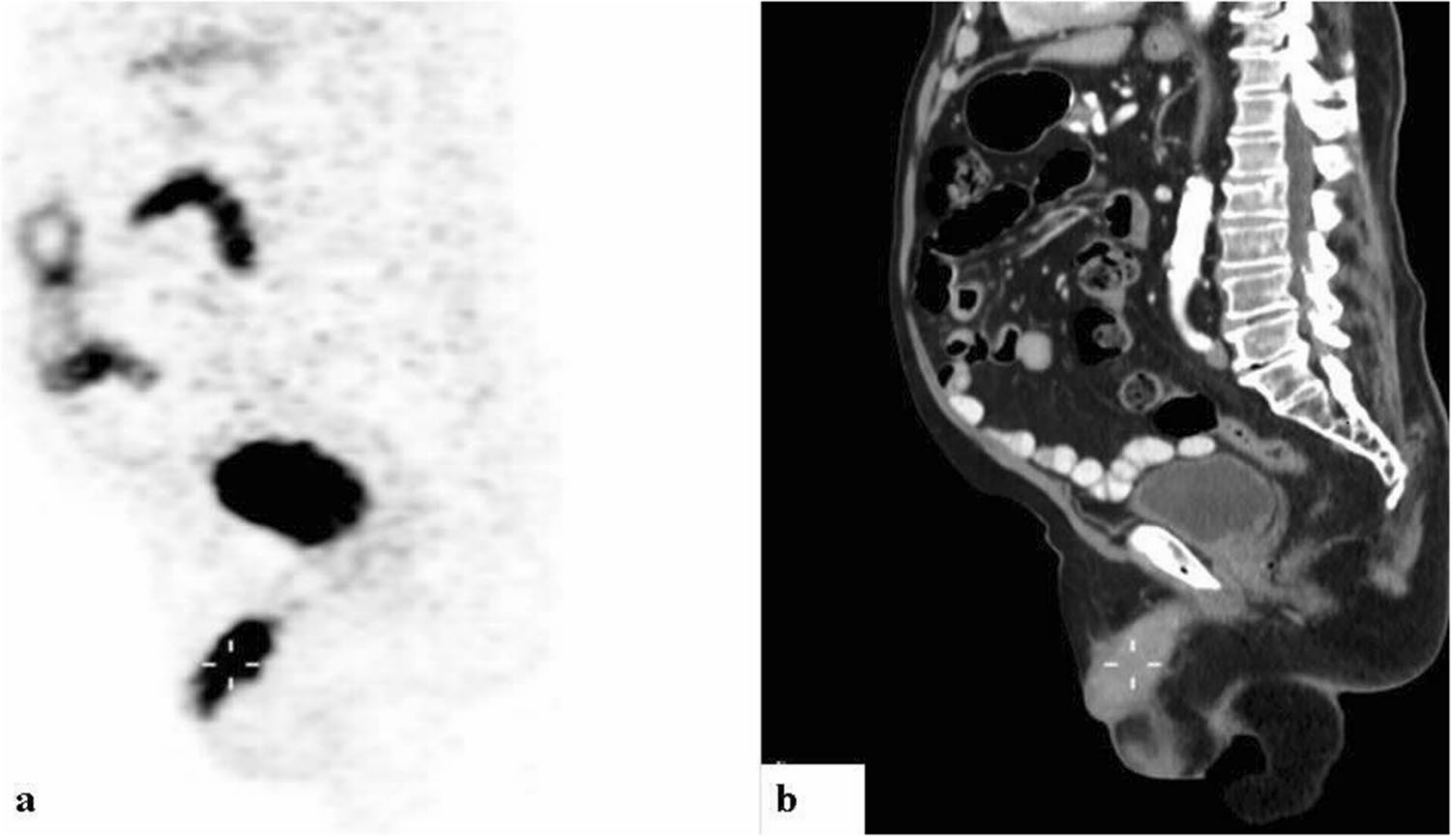
Ga-68 PSMA-PET (**a**) and CT (**b**) sagittal slices demonstrating diffusely increased uptake along the penis. A 71-year-old man with metastatic prostate cancer of neuroendocrine differentiation, in the absence of prior prostatectomy, with a Gleason’s score of 7 and a PSA of 3 (patient #5, Table 1).

**Figure 2:**
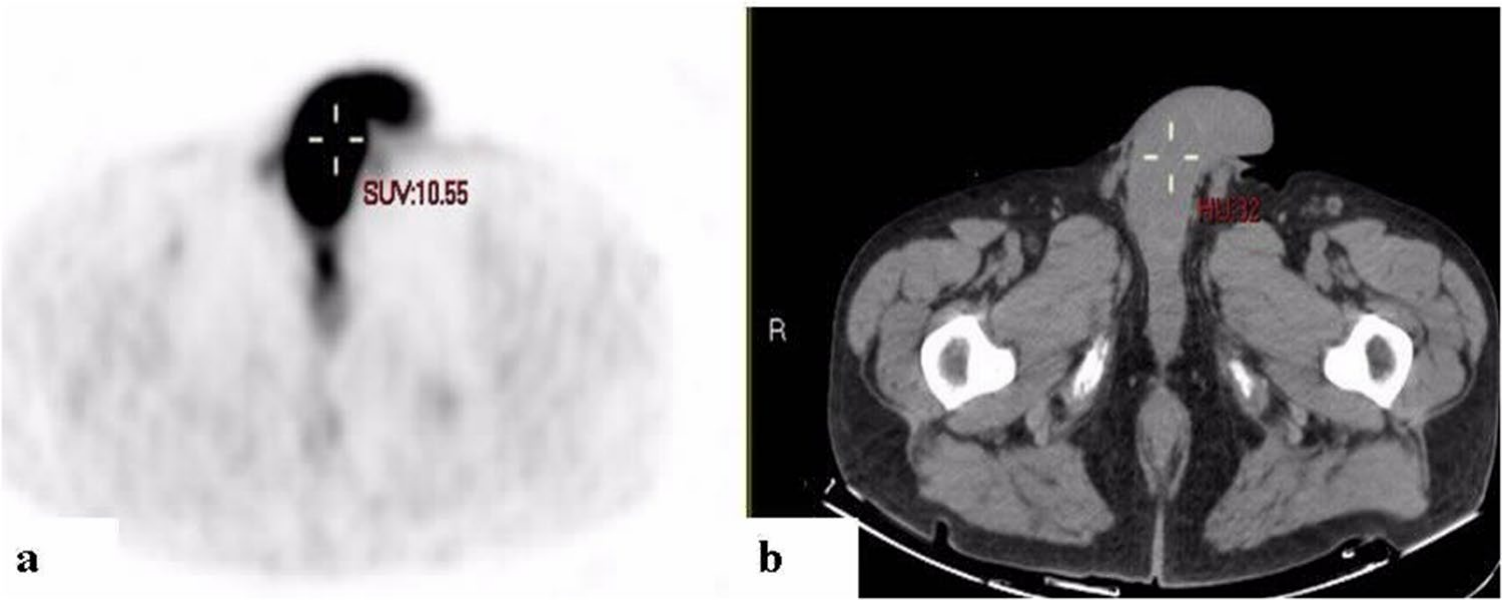
18F-FDG PET (**a**) and CT (**b**) axial slices demonstrating diffusely increased uptake along the penis. A 73-year-old man with relapse of diffuse large B-cell lymphoma (patient #16, Table 1).

**Figure 3:**
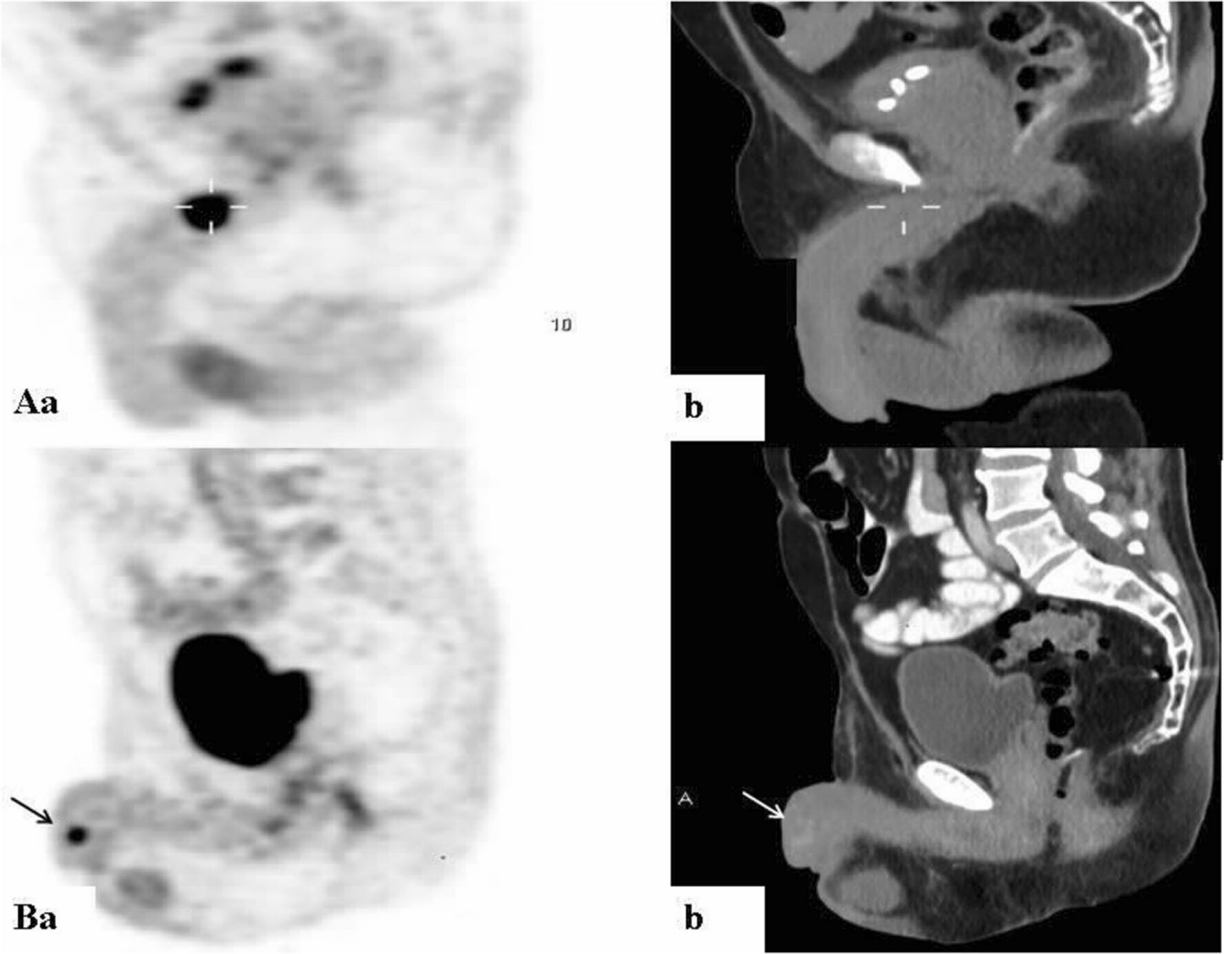
18F-FDG scans demonstrating focally increased uptake at different places along the penis on PET (**a**) and CT (**b**) sagittal slices. (**A**) An 84-year-old man with metastatic high-grade urothelial carcinoma (patient #22, Table 1). **(B**) A 49-year-old man with metastatic adenocarcinoma of the lung (patient #20, Table 1).

**Figure 4:**
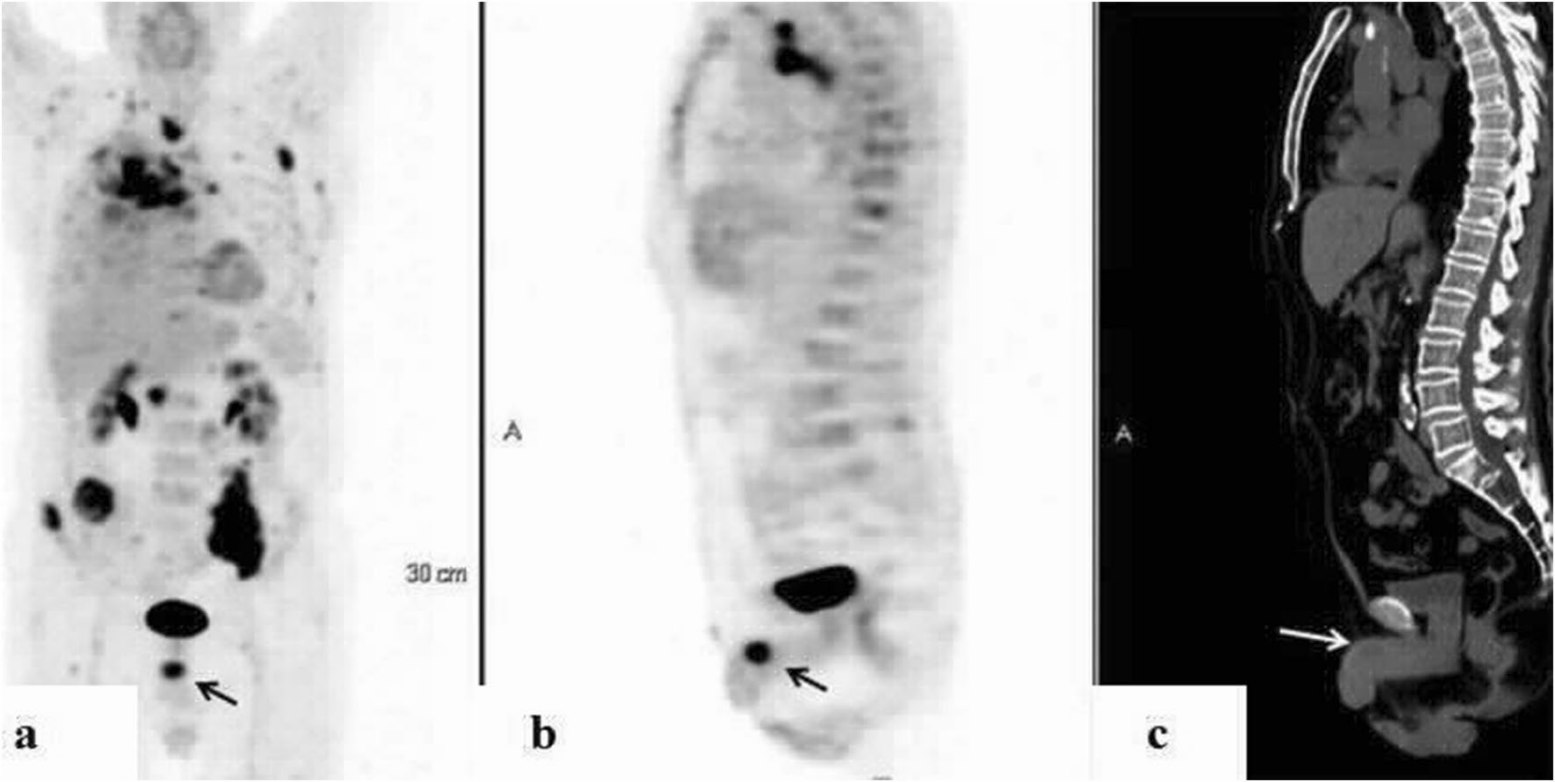
18-FDG PET/CT: maximum intensity projection (MIP) (**a**) representative PET (**b**) and CT (**c**) axial slices demonstrating focally increased uptake in the penis. A 79-year-old man with metastatic non-small cell lung carcinoma and a prior history of prostate cancer who had undergone prior transurethral resection of the prostate (patient #21, Table 1).

**Figure 5:**
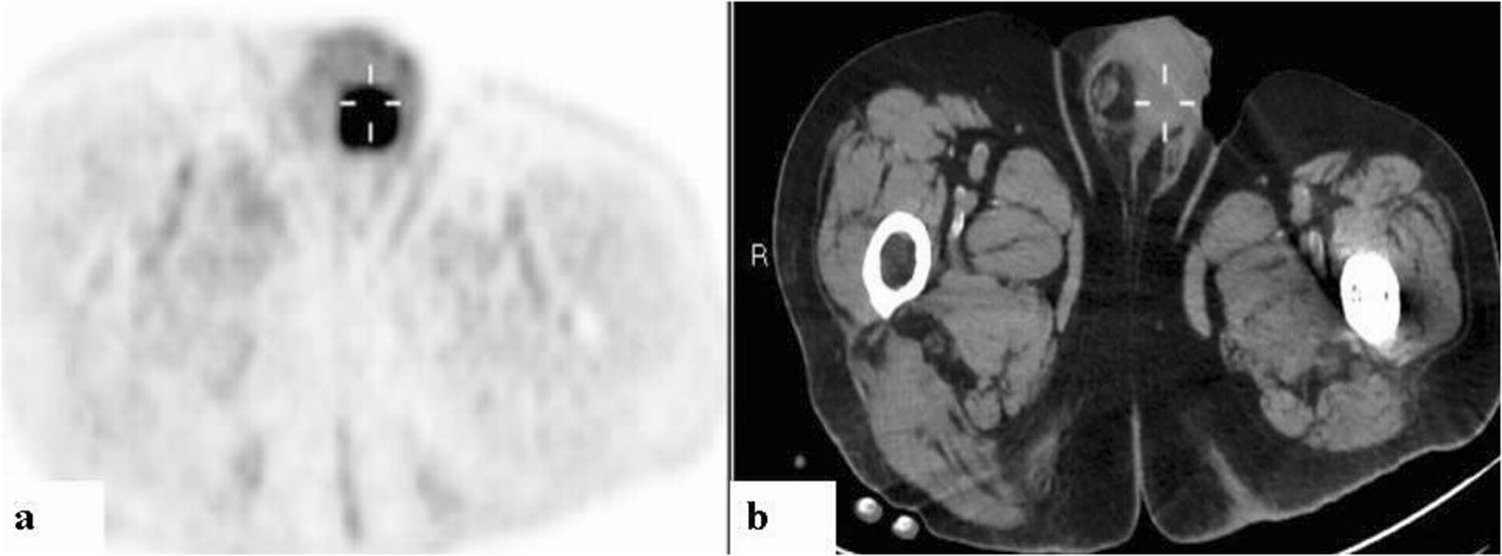
18F-FDG PET (**a**) and CT (**b**) axial slices demonstrating focally increased uptake at the root of the penis. An 80-year-old man with metastatic high-grade pelvic leiomyosarcoma (patient #23, Table 1).

For the entire cohort, uptake along the penile lesion was focal in 13 (54.2%) and diffuse in 11. Among the 12 men with prostate cancer, uptake in the penis was diffuse in 7 (58.3%) (Fig. 1) and focal in 5. Among the men with non-prostate primary cancer, enhancement was diffuse in one of the 3 with CRC, and in 3 of the 4 with DLBCL (Fig. 2). Increased uptake was focal in the remaining 2 patients with CRC; in one with DLBCL; in the 2 with NSCLC; and in the patients with UC, pelvic leiomyosarcoma and AML (Figs. 3, 4, and 5). No correlation was observed between cancer type and the pattern of tracer uptake (*p* = 0.680), or between the occurrence of previous prostatectomy in prostate cancer patients and the tracer pattern (*p* = 0.567).

##### The location and size of focal PSL

Among the men with primary cancers other than the prostate, 8 had penile lesions with focal uptake. In the 2 men with CRC and focal enhancement, the lesion was present at the penile root. Similarly, for those with UC, AML, and pelvic leiomyosarcoma, penile enhancement was seen at the root. In the 2 men with NSCLC, penile enhancement was localized along the shaft. In the patient with DLBCL and focal tracer uptake, the penile lesion was focused at the glans.

**Figure 6:**
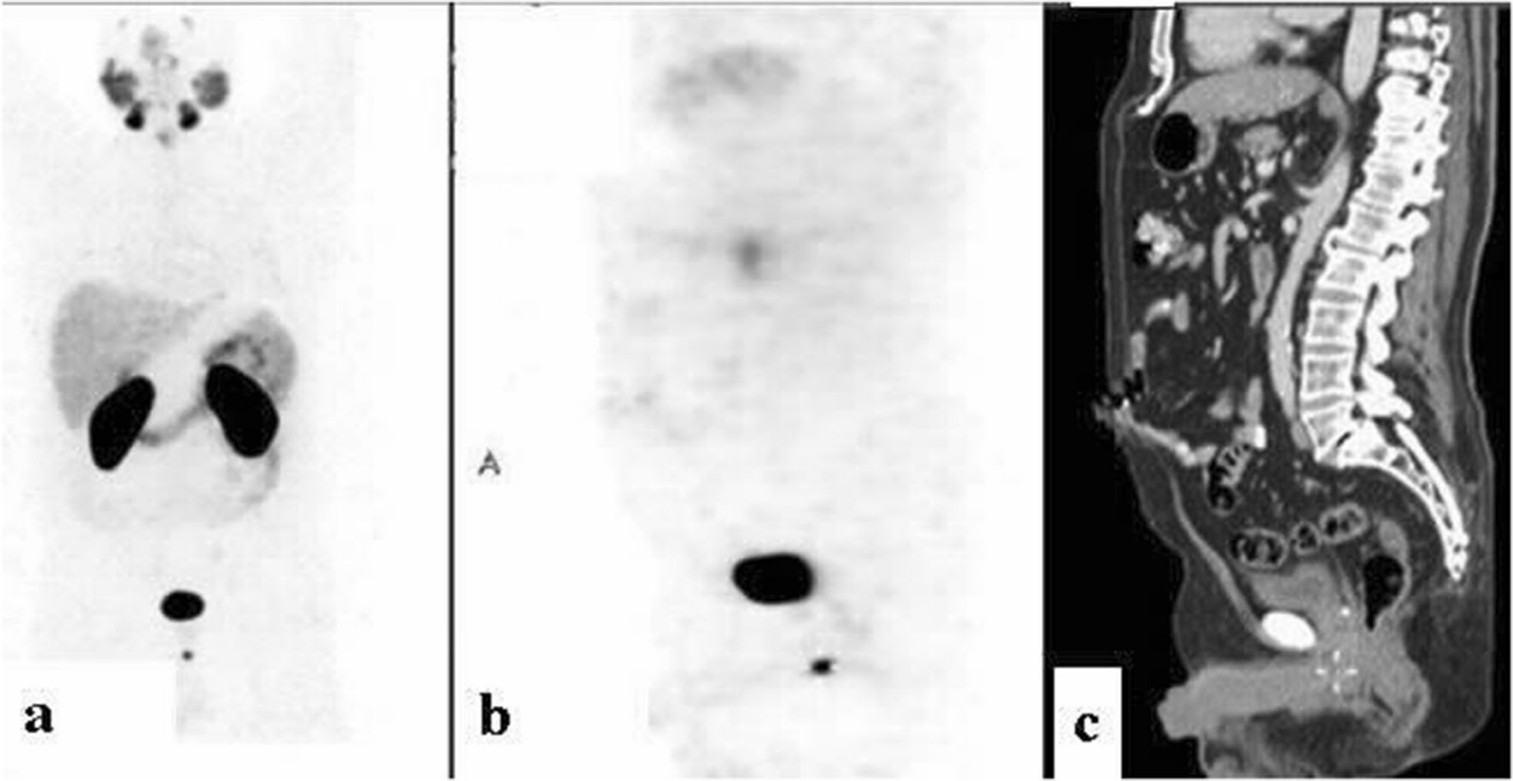
Ga-68 PSMA PET/CT: maximum intensity projection (MIP) (**a**) representative PET (**b**) and CT (**c**) axial slices demonstrating focally increased uptake in lesions at the root of the penis. A 62-year-old man with metastatic adenocarcinoma of the prostate with perineural invasion, who had not undergone prior prostatectomy, with a Gleason’s score of 7 and PSA of 76 (patient #6, Table 1).

Among the 5 men with prostate cancer and focal enhancement, the lesion was visible at the penile root in 3 (Fig. 6). In the other 2, focal enhancement was present along the penile shaft.

In the 14 men with focal penile lesions, diameter length ranged from 10 to 30 mm, with a mean of 16.5 ± 6.8 mm. In the 5 men with prostate cancer and focal lesions, the diameter length ranged from 10 to 17 mm, with a mean of 12.2 ± 2.9 mm.

#### Penile changes on CT

**Figure 7:**
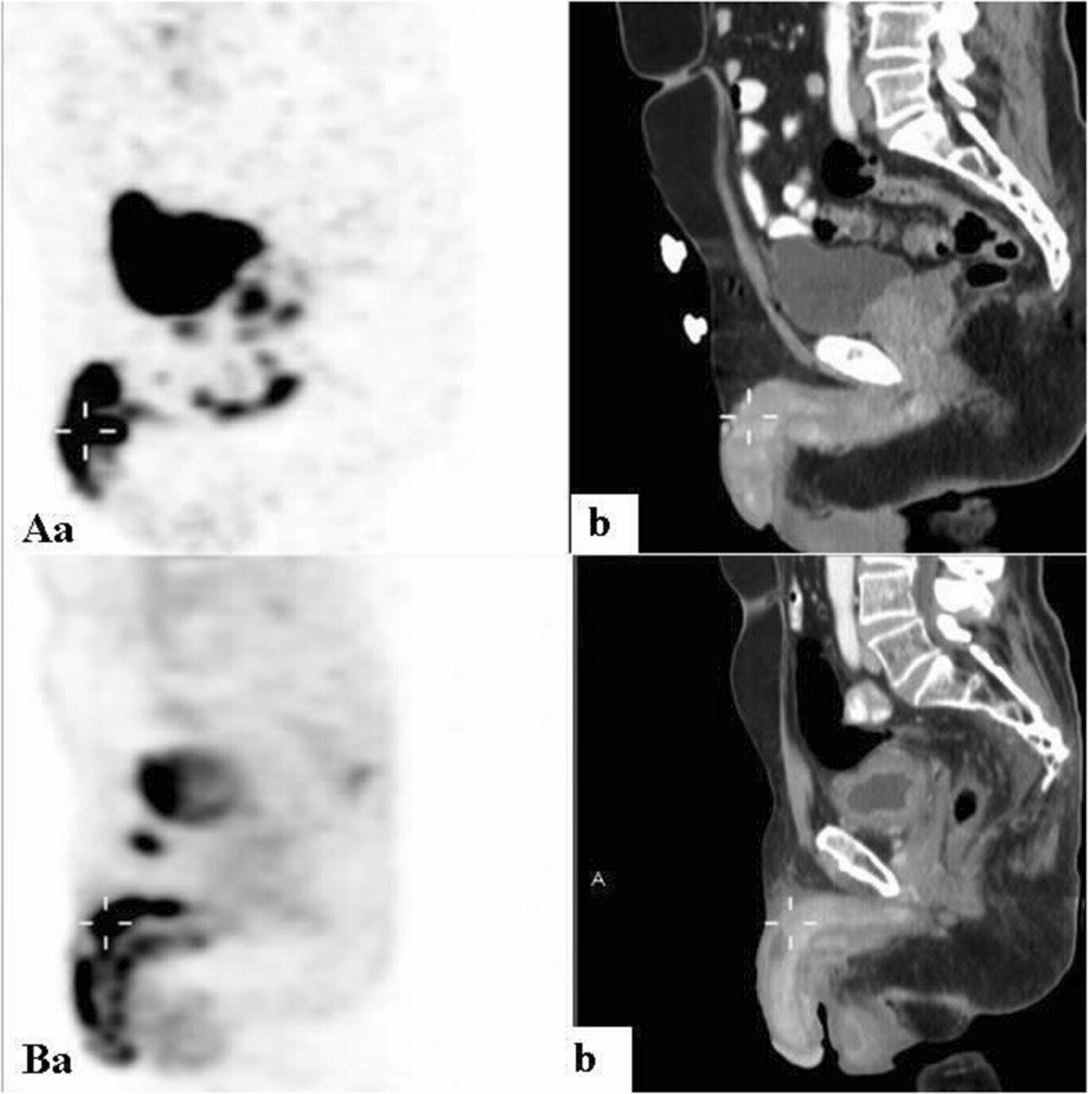
Diffuse non-homogeneous increased penile uptake on PET (**a**) with corresponding soft tissue lesions infiltrating the penis on CT (**b**) sagittal slices of: (**A**) A Ga-68 PSMA scan of a 68-year-old man with metastatic adenocarcinoma of the prostate, who had undergone robot assisted laparoscopic radical prostatectomy, with a Gleason’s score of 8 and PSA of 4.3 (patient #3, Table 1). (**B**) An 18F-FDG scan of a 72-year-old man with metastatic adenocarcinoma of the colorectum (patient #17, Table 1).

In 15 (62.5%) of the men, CT imaging showed loss of the typical penile texture. Of the 12 with prostate cancer, nine (75%) had corresponding CT findings. Penile changes on CT were also visible in 2 of the 3 men with CRC, one of 2 with NSCLC, one of 3 with DLBCL, the man with UC, and the man with pelvic leiomyosarcoma (Fig. 7). For the 9 remaining patients, CT did not demonstrate clear corresponding findings on CT.

Of the 15 patients with penile changes on CT, thirteen had hyperdense lesions: 7 with diffuse heterogeneous soft tissue mass infiltration along the penis and 6 with a focal soft tissue mass. Penile lesions appeared hypodense on CT in one man with prostate cancer and one with NSCLC.

#### Extra-penile lesions

##### Invasion of the pelvic LNs

PET/CT revealed Ga 68-PSMA/ 18F-FDG-avid findings in the pelvic LNs in 20 (83.3%) men. Pelvic lymphadenopathy was detected in 10 (83.3%) men with prostate cancer; three of the 4 with DLBCL; two of the 3 with CRC; the 2 with NSCLC; and in those with UC, pelvic leiomyosarcoma, and AML.

##### Additional metastatic sites

In 22 (91.7%) men, PET/CT revealed lesions with enhanced uptake, suspicious for metastases, in sites other than the penis or pelvic LNs (Table 2). Bone lesions were most common, present in 13 (54.2%) men. The second most common site for enhancement was the liver, in 6 (23.1%) men, followed by the bladder with discrete supraphysiological uptake in 5 (20.8%) men.

**Table 2:** Sites of extra-penile pathological findings on PET/CT.

Primary cancer type	Site(s)
Prostate	Liver, vertebrae
	b/l lungs, bony pelvis
	Bladder, liver, ribs
	Bladder, pelvic muscles, vertebra
	Mediastinal and retroperitoneal LNs
	Bony pelvis, rt scapula, sternum
	Bladder, liver, ureter, urethra
	b/l lungs, rt clavicle
	rt sacrum
	Bladder, liver, ribs, vertebrae
	rt retroperitoneal LNs, mediastinal LNs
Lymphoma	Adrenals, b/l humerus, brain, prostate, ribs, rt kidney, seminal vesicles, skull, spleen, sternum, supra- and infra-diaphragmatic LNs, testicles
	Paranasal sinuses, optic nerve
	lt bony pelvis, testicles
	Liver, lungs, adrenal
Colorectal	Bony pelvis, lt lung, liver
	Bladder, pelvic floor muscles
Lung	Retroperitoneal LNs
	Adrenal, bony pelvis, b/l iliac veins, LNs of the thorax, pancreatic head, ribs, vertebra
Bladder	Seminal vesicles, prostate
Pelvic sarcoma	Testicles, seminal vesicles
Acute myeloid leukemia	Bladder, soft tissue of the thoracic cavity, vertebrae

*b/l* bilateral, *rt* right, *lt* left, *LN* lymph nodes.

PET/CT detected metastatic lesions, outside the penis and LNs, in 11 (91.7%) of the 12 men with prostate cancer. Bony lesions were also the most frequent site of secondary lesions among the men with prostate cancer, found in 8 (66.7%), followed by the liver and bladder, each identified in 4 (33.3%) patients.

The involvement of neighboring soft tissue structures within the pelvis was noted in 4 men, each with non-prostate cancers. Increased uptake in the seminal vesicles and testicles was observed in one man with DLBCL and in the patient with pelvic leiomyosarcoma, in the testicles of another patient with DLBCL, and in the seminal vesicles and prostate in the man with UC.

### Additional imaging and lab findings

#### Penile findings on ultrasound and MRI

Penile ultrasound findings were available for three men. For one, a patient with prostate cancer with diffuse penile enhancement on PET/CT, ultrasound was helpful in revealing diffuse masses bilaterally along the superficial penis and along the corpus cavernosum. For the other 2, however, ultrasound did not help delineate the penile lesions seen on PET/CT. For these 2 patients, one with prostate cancer and diffuse uptake on PET and the other with undifferentiated NSCLC with focal uptake along the shaft, ultrasound of the penis and scrotum was normal, with intact Doppler flow. In one patient with prostate cancer, a penile lesion was detected on MRI, which corresponded to the PET/CT finding.

#### PSA values

For 11 (88%) of the men with primary prostate cancer, a PSA value was available at the time the penile lesions were revealed by PET/CT. The PSA values ranged from 0.008 to 83 ng/mL, with a mean of 25.1 ± 30.2 (normal values vary by age and ethnicity, though are generally considered to be ≤ 4 ng/mL).

### Clinical follow up

#### Time lapsed from diagnosis of the primary cancer

Ten (41.7%) of our patients had prior PET/CT studies in our system, none of which revealed PSL. The duration of time from the initial diagnosis of primary malignancy to the finding of PSL on PET/CT was available for 23 men. Among these men and among the subset of 11 with primary prostate cancer for whom this information was accessible, the length of time from diagnosis to PSL identification ranged from less than 1 year to 16 years, with a median of 4 years.

PSL were not detected in any of the men during initial staging. In 19 men, the first observed penile findings were detected on PET/CT scans performed during relapse of the primary cancer. In the remaining 5, the scans were done as part of monitoring of known advanced disease over the course of the first year following diagnosis: three men with prostate cancer, one with CRC, and one with DLBCL.

#### Imaging follow-up

For 11 (48.5%) men, follow-up PET/CT studies were performed between one and 40 months following the initial study that showed PSL. Four were done in men with prostate cancer using PSMA and 7 were FDG scans from men with other primary malignancies. In the 4 with prostate cancer, PSL were more pronounced in 3; while in one, the lesion decreased in size in parallel with treatment. Among the others with repeat scans, three had CRC; these showed more pronounced uptake in one, a widening of the lesion in another, and without apparent change in the third. PSL appeared stable on follow-up in the patient with AML. In the man with DLBCL and the man with NSCLC, the PSL enlarged. In the patient with pelvic leiomyosarcoma, PSL decreased in size with treatment.

#### The time lapsed from the first finding of penile lesions on PET/CT to death

The occurrence of death during the study period was known to us for 15 (62.5%) of the men in our cohort. Chart review revealed that each of these patients had died as a result of complications related to their malignancies. Among those who died, the survival time from the first scan that demonstrated PSL ranged from one to 48 months, the median was 8 months. In men with prostate cancer, proof of death during the study period was available for 6 (50%), with survival ranging from one to 48 months, with a median of 11.5 months. The other 9 who died included the 2 with NSCLC, the man with UC, three of those with DLBCL, two with CRC, and the patient with AML.

## Discussion

This study reviewed all PSL identified by the PET/CT scans performed in our medical center over an 8-year period. To the best of our knowledge, this is the first case series to describe PET/CT findings of PSL in a relatively large group of patients with diverse primary malignancies. We found evidence of PSL in 0.13% of all male patients who had PET/CT scans performed for evaluation of known or suspected oncological diseases, and in 0.61% of those with suspected or confirmed prostate cancer who had undergone Ga 68-PSMA studies.

While the rate of PSL among all cancers is unknown, the reported incidence among men with prostate cancer ranges from 0.1%, as described by a recently published paper by an Australian group that identified 5 men with prostate cancer using PSMA-avid PSL, to 0.5%, based on an older autopsy study^21,22^. Cancer of the prostate was the primary cancer for half our cohort; and together with cancers of the colorectum, and bladder, these comprised two-thirds of the primary cancers. However, we also detected PSL in patients with NSCLC, DLBCL, AML, and pelvic leiomyosarcoma. PSL have previously been recognized in lung cancer, lymphomas, AML, and pelvic leiomyosarcoma, though more than 90% originate in cancers of the genitourinary and gastrointestinal tracts^23–26^. Our 4 patients with lymphoma had DLBCL, corroborating reports that while found in both T- and B-cell lymphomas, PSL appear most commonly in DLBCL^27,28^.

On PET, we observed a mean SUVmax of close to 8 in the uptake of PSL, with no correlation between cancer histology and the degree of tracer uptake. The pattern of the PSL was focal in just over half, and diffuse in the remaining cases, without a correlation between cancer type and pattern of uptake. Prior studies reviewing PSL on PET have illustrated similar findings, with a mean SUVmax of 7.8 according to one, and with PSL appearing both focally and diffusely^2,4,21,29^. We found no association between a history of prostatectomy and the degree or pattern of uptake. Seeding of cancer cells into the urethra has been theorized to occur during TURP, though some have postulated that this is highly unlikely. To the best of our awareness, no associations have been found between the method of prostate resection and penile metastases^5,30^.

Among our patients with focal uptake, PSL localized most commonly to the root in three fifths, to the shaft in almost one third, and to the glans in only one patient with DLBCL. PSL was previously demonstrated at the root and shaft at equivalent rates, and at the glans in one quarter of the cases^9,25,31^. Uptake along the shaft in our 2 patients with NSCLC strongly implied vascular spread. In the 4 men with involvement of adjacent pelvic soft tissue structures, who were all with penile uptake that was either diffuse or localized to the base, PSL likely resulted from local invasion. In the rest of our patients, however, such inferences were more difficult to draw. While the route of dissemination is generally uncertain, enhancement at the shaft or glans has been cautiously suggested to imply cancer invasion by venous spread; and enhancement at the root is more likely to imply local invasion^6^.

CT contributed to the characterization of 63% of the PSL detected on PET, as exhibited by hyperdense focal soft tissue mass, diffuse soft tissue infiltration, or hypodensity. For the remaining PSL detected in PET, we did not find corresponding lesions on CT, and specifically not in 4 of our 5 patients with lymphomas and leukemia. While the nonappearance of penile changes on CT may be explained by the absence of contrast media during most of these exams, it underscores the value of PET in revealing PSL. Other studies have also reported the lack of penile findings on the CT portion of PET/CT^32^. PSL persisted in 82% of our patients who had follow up scans, and decreased in size in only 2 patients, in concurrence with treatment. This corroborates the evidence that lesions reflect PSL. Likewise, lesion persistence on repeat PET/CT in the aforementioned paper also demonstrated its consistency with PSL^32^.

Seventy percent of PSL in the current study were detected in patients in their 7th and 8th decades, and only one patient was younger than 50 years. Similarly, the mean age of PSL presentation in other reviews ranges between 60 and 80 years^5^. PSL were identified early in the disease course in some of our patients with extensive disease at presentation, and up to 16 years following diagnosis in the context of relapsed cancer. In more than 90% of our patients, extra-penile metastases were evident on PET/CT, illustrating the advanced nature of the disease. Prior reviews have illustrated that penile lesions mostly emerge years into the course of disease, though in rare instances, they present very early. Moreover, though PSL generally emerge in the setting of disseminated disease, they have also been described in the absence of other metastases^5,33^. In addition, we found PSL in patients with a wide range of PSA values, as has been previously demonstrated^34^.

We observed a mean survival rate of 13.3 months, and more than half died within 6 months following PET/CT detection of PSL. The median survival was 3.5 months longer in those with prostate cancer than with other malignancies. Likewise, others reported a mean survival of 14 months in those with PSL, with few surviving more than 2 years, and with poorer prognosis in those with cancers of non-urological origin^3,15^.

Our study has several limitations, including its retrospective nature and restricted sample size, and its being conducted at a single institution. PSMA was only introduced at the midpoint of the study, yet it appears to be a much more sensitive tool than FDG for the detection of metastatic lesions in men with prostate cancer, particularly at the penis. Furthermore, the prevalence calculated for PSL among cancer patients and prostate cancer patients alone are likely understated because they are based on the total number of FDG and PSMA PET/CT studies for suspected and confirmed malignancies, and we could not distinguish between those with definite cancers and in particular, those with disseminated disease. Finally, a pathological report was available for only one of the penile lesions seen on imaging. Notably, all 24 of our patients had advanced disease and the penile lesions were indicative of metastatic spread. For these reasons, and due to the exceeding rarity of primary penile cancer, there was no clinical need for pathologic correlation at this stage as has been reported elsewhere, and this was decided against by the treating clinicians^35^.

Despite these limitations, we were able to detect pathological PET/CT penile lesions in a series of 24 patients with a variety of malignancies. Even though we could not correlate these findings morphologically, careful characterization of penile findings using the dual modality of PET/CT with clinical correlation and evaluation of follow-up studies strongly support the postulation that the described PET/CT lesions of the penis represent PSL. Larger studies are warranted to assess the clinical implications that emerge from identification of PSL.

## Conclusion

In malignancies with avidity for FDG and PSMA, PET/CT enables the detection of PSL. Our study confirms their rarity, though likely understates the true prevalence. Detecting PSL is important because these represent a possible metastatic site in a range of cancers. Moreover, PSL contribute to morbidity and portend a poor prognosis, and appropriate treatment measures must be instituted promptly. Accordingly, PET/CT is a critical instrument for assessment of the male genitalia, especially in men with advanced disease and for whom relapse is a concern.
